# The Management of Metastatic Castrate-Sensitive Prostate Cancer: From Guidelines to Real-World Practice

**DOI:** 10.1093/oncolo/oyad047

**Published:** 2023-04-04

**Authors:** Georges Gebrael, Vinay Mathew Thomas, Umang Swami, Neeraj Agarwal

**Affiliations:** Division of Oncology, Department of Internal Medicine, Huntsman Cancer Institute, University of Utah, Salt Lake City, UT, USA; Division of Oncology, Department of Internal Medicine, Huntsman Cancer Institute, University of Utah, Salt Lake City, UT, USA; Division of Oncology, Department of Internal Medicine, Huntsman Cancer Institute, University of Utah, Salt Lake City, UT, USA; Division of Oncology, Department of Internal Medicine, Huntsman Cancer Institute, University of Utah, Salt Lake City, UT, USA

## Abstract

This commentary highlights 2 international studies on real-world treatment trends and patterns among patients with metastatic castration-sensitive and resistant prostate cancer and how interventions targeting physicians and patients can bridge the gap between evidence-based medicine and real-world practice.

The management of metastatic castrate-sensitive prostate cancer (mCSPC) has undergone a revolution in the past decade. Currently, several agents are added upfront to androgen deprivation therapy (ADT). This approach, referred to as treatment intensification, is considered the pillar of mCSPC treatment. Upfront docetaxel,^1^ abiraterone,^2,3^ apalutamide,^4,5^ enzalutamide,^6,7^ and recently the combination of docetaxel with either darolutamide^8^ or abiraterone^9^ have all led to better overall survival (OS) in their respective landmark phase III clinical trials. Interestingly, ADT intensification did not negatively impact the quality of life. For example, in the TITAN trial, adding apalutamide to ADT neither increased fatigue nor affected the health-related quality of life as assessed by standardized tests, such as brief fatigue inventory and functional assessment of cancer therapy-prostate (FACT-P).^10^ Therefore, many oncologic societies worldwide have changed their practice guidelines from ADT monotherapy to recommend treatment intensification as the new standard of care (SOC). In the US, the upfront use of the agents mentioned above, except docetaxel alone, is considered as Category 1 per the National Comprehensive Cancer Network “NCCN” guidelines 2023.^11^ Similarly, in Europe, upfront enzalutamide, abiraterone, apalutamide, and docetaxel are recommended with the highest level of evidence [I,A] per the European Society of Medical Oncology clinical guidelines 2020.^12^ Moreover, prescribing ADT alone is discouraged, to be used solely in frail patients or when intensification therapy is clearly contraindicated.^12^ In light of these practice-changing data and strong recommendations by guideline committees across the world, it is clear now that treatment intensification is the first and foremost treatment of mCSPC and should be adopted widely by practitioners in the real-world practice setting.

In this context, Barata et al. performed 2 retrospective studies across 5 European countries (France, Germany, Italy, United Kingdom, and Spain) and the US.^13,14^ They included adult men classified, at the time of data collection, between January and June 2020, as either mCSPC or previously mCSPC who progressed to metastatic castrate-resistant prostate cancer (mCRPC). The authors assessed mCSPC treatment between 2016 and 2020. One thousand five hundred sixty men with mCSPC were included, of whom the majority (*n* = 1321) were European. During the 2019-2020 period, ADT ± first-generation antiandrogens use ranged from 33% to 61% across European countries. Intensified therapy in the same countries and during the same period ranged from 44% to 68%. In the US, although 59% of patients receiving intensified ADT (49% novel hormonal therapy (NHT) and 10% taxane ± NHT) as of 2019-2020, 39% of patients still received ADT ± first-generation antiandrogens, which was no longer the SOC at that time. Across all the included countries, the comparison of the 2016-2018 and 2019-2020 periods showed a trend toward decreasing use of ADT ± first-­generation antiandrogens with a simultaneous increase in the use of ADT intensification therapy. The investigators concluded that in a real-world mCSPC setting, despite an uptake of intensified ADT regimens, a significant number of patients across Europe and the US are still receiving suboptimal treatment, ie, ADT without intensification. In their second paper, Barata et al. highlighted the overlap between the agents increasingly used in the mCSPC setting and mCRPC, which creates an unmet need for effective treatment options after the onset of mCRPC. Their study objective was to assess whether the agent used in the mCSPC setting affects the subsequent first-line (1L) treatment pattern in the mCRPC setting. The study included 722 real-world adult patients diagnosed with mCRPC at the time of data collection and the receipt of the first-line (1L) therapy in the mCRPC setting between 2016 and 2020 across countries from Europe and the U.S. NHT was the most common 1L mCRPC treatment in both Europe and the U.S. (65% and 75%, respectively). In the European cohort, among patients who received upfront NHT in the mCSPC setting, 47% received docetaxel, and 22% received another NHT in the 1L mCRPC setting. For the remaining patients who received ADT monotherapy, or ADT plus docetaxel in the mCSPC setting, an NHT was the most used agent subsequently. In the US, NHTs were the most common agents used in 1L mCRPC, regardless of the treatment received in the mCSPC setting, whether it was ADT alone or intensified with docetaxel or an NHT. However, the NHT received in the mCRPC setting differed from the one already prescribed in mCSPC. The authors concluded that physicians are ­considering previous treatment when prescribing 1L therapy in an mCRPC setting.

The findings of suboptimal use of intensified ADT by Barata et al. are similar to those previously reported across several studies performed in the real-world US population (Table 1). Swami et al. reported that 56% of the patients with mCSPC received ADT alone between January 2014 and June 2019. This study included 4221 adult patients with mCSPC with either commercial insurance or Medicare plans collected from Optum Health Insurance Claims Database. Even in 2019, more than half of the patients (52.6%) received ADT alone. Likewise, patients with aggressive tumor features, such as visceral metastasis, received intensified ADT in only 26.6% of cases.^15^ In a smaller set of patients, George et al. showed that more than 50% of patients received non-level I evidence-based treatment as of 2019, such as ADT alone or in combination with first-generation antiandrogens or other treatments. Noteworthy, no significant treatment difference between Black and White men was reported in this study.^16^ In a retrospective US real-world study performed by Ryan et al., which included 6517 patients from Optum’s de-­identified Clinformatics Data Mart Database from January 1, 2014, to July 31, 2019, and 13 324 patients from Medicare Fee-for-Service from January 1, 2014, to December 31, 2017, 45% and 46% of patients received ADT monotherapy, 7% and 1% received upfront abiraterone acetate, and 6% and 2% received upfront docetaxel, respectively.^17^ Another study presented at the 2021 American Society of Clinical Oncology (ASCO) annual meeting by Freedland et al., which included 35 195 patients from the Medicare database, showed that less than 25% of patients received intensified ADT, and around 68% of patients received ADT alone in 2018. African American men in this cohort received less ADT plus NHT than White or Hispanic men.^18^ In 2022, Freedland et al. reported that 69% of patients received ADT ± first-generation antiandrogens between 2018 and 2021.^19^

In conclusion, a progressive and steady trend toward increased ADT intensification was observed over recent years. However, an unmet need still exists to identify the reasons behind the underutilization of ADT intensification therapy and a need to push forward adherence to the guidelines-­recommended regimens supported by the level-1 evidence in the overall population. Interventions targeting physicians and patients can bridge the gap between ­evidence-based medicine and real-world practice. According to Freedland et al., the top 5 reasons why physicians do not choose treatment intensification in an mCSPC setting are concerns about drug tolerability (38%), perceived lack of clinical trial evidence of overall survival improvement (31%), lack of reimbursement (26%), patient financial constraints (20%), and lack of clarity about utilizing NHTs earlier versus later in disease (21%).^19^ Of note, the latest ASCO data projected a shortage of 2258 oncologists in the US because of growing oncologic demand by 2025.^20^ This oncology providers’ shortage means that the existing workforce will be more likely to see a higher number of patients daily, managing several cancer subtypes, thus reducing their capacity to stay updated on the latest guidelines for each cancer type. Therefore, improving education about the latest data among the providers will be critical and may be enhanced through easily accessible platforms such as social media, hybrid conferences, and webinars. Besides physician education and awareness, one major reason behind the underutilization of intensified ADT treatment is financial. Joyce et al. recently reported that the mean treatment-related out-of-pocket cost in the first year was $165 for ADT monotherapy, whereas it is $4236 for upfront intensification with an NHT.^21^ Based on these data, efforts by stakeholders (ie, insurance companies, pharmaceutical companies, and healthcare institutions) are a must to reduce the financial burden and widen the use of intensified therapy to a more extensive set of patients. The availability of these novel therapies in rural and local areas without the necessity to travel elsewhere can also reduce financial toxicity. With NHT being used earlier in prostate cancer treatment history, concerns regarding the efficacy of docetaxel or NHT in mCRPC after prior NHT use in an mCSPC setting should also be addressed in future prospective trials. Interestingly, in a US-based study among 100 patients with metastatic prostate cancer, 21% of patients did not agree with “I rely on my doctor to tell me how to treat my prostate cancer.”^22^ Raising awareness and education among patients about evidence-based treatment is as vital as doing this among physicians. Educating patients about the treatment’s survival outcomes benefits, impact on quality of life or pain relief, side effects, and cost-­effectiveness could positively impact cancer care and prognosis. This knowledge of patients and patient advocates can be enhanced through open-access patient-friendly publications, social media platforms, webinars, or open-access websites such as “www.cancer.net,” which is the ASCO’s patient information website, or through conference talks addressed exclusively to patients.^23^ Eventually, the involvement of all stakeholders will be the key to improving the utilization of evidence-based medicine and, subsequently, the survival outcomes in our patients with metastatic prostate cancer.

**Table 1.** Summary of the major studies evaluating treatment patterns in the mCSPC setting in the US.

Study Name	Total number of participants	Data source	Differential percentage of therapies
Barata et al.^13^	1560 (*N* = 239 in the US)	Adelphi prostate cancer disease specific programme	2019-2020 (In the US) ADT alone ± NSAA: 39% ADT + docetaxel: 10% ADT + NHT: 49%
Swami et al.^15^	4221	Optum health insurance claims database (includes patient claims data from both commercially insured and Medicare advantage populations)	2019 *N* = 614 ADT alone: 52.6% ADT + NSAA: 15.3% ADT + docetaxel: 7.6% ADT + NHT: 22.6% ADT + docetaxel + NHT: 1.8%
George et al.^16^	858	ConcertAI oncology dataset (a community oncology dataset)	2019 ADT alone: 26.6% ADT + NSAA: 16.5% ADT + docetaxel: 10.1% ADT + NHT: 34.2%
Ryan et al.^17^	13 324	Medicare fee-for-service	2014-2017 ADT monotherapy: 45.8% ADT + abiraterone: 0.8% ADT + docetaxel: 1.6%
Ryan et al.^17^	6517	Optum’s de-identified Clinformatics Data Mart Database (commercial insurance/Medicare advantage)	January 2014–Jul 2019 ADT monotherapy: 45.4% ADT + abiraterone: 7.2% ADT + docetaxel: 5.6%
Freedland et al.^18^	35 195	Medicare database (parts A, B, and D)	2018 (White-non Hispanic) *N* = 3537 ADT alone: 68.2% ADT + NSAA: 10.1% ADT + docetaxel: 5.3% ADT + NHT: 16.5%
Freedland et al.^19^	621	Multiple US academic/community ­practices	July 2018-November 2021 ADT alone ± NSAA: 69% ADT + NHT: 26% ADT + chemotherapy: 4%

Abbreviations: ADT, androgen deprivation therapy; NHT, novel hormonal therapy; NSAA, non-steroidal anti-androgens.
